# Using occupancy modeling to provide insights into suitable habitat characteristics for the already restricted and critically endangered Olalla's titi monkey (*Plecturocebus olallae*)

**DOI:** 10.1007/s10329-024-01171-3

**Published:** 2024-11-29

**Authors:** Jesús Martínez, Robert Márquez, Ariel Reinaga, Marco Campera, Vincent Nijman, Robert B. Wallace

**Affiliations:** 1 Wildlife Conservation Society, Edificio Torre Soleil, #987 Calle Jaime Mendoza, San Miguel, La Paz, Bolivia; 2 Red Boliviana de Primatología (RedBolPrim), La Paz, Bolivia; 3 Andean Bear Conservation Alliance, Cleveland, USA; 4 Cornell University, Fernow Hall, 226 Mann Dr, Ithaca, NY 14853 USA; 5 School of Biological and Medical Sciences, Oxford Brookes University, Oxford, UK; 6 School of Law and Social Sciences, Oxford Brookes University, Oxford, UK; 7 Wildlife Conservation Society, 185th Street and Southern Boulevard, Bronx, NY 10460 USA

**Keywords:** Habitat restriction, Conservation, Threats, Fragmented forests, Occupancy modeling

## Abstract

Knowledge about changes in wildlife populations over time is essential for making informed decisions regarding their conservation. We evaluated the influence of distinct habitat factors on the occupancy of Olalla's titi monkey (*Plecturocebus olallae*), a Critically Endangered primate endemic to Bolivia. We assessed the presence of titi monkey groups using the playback technique, employing point counts in 582 quadrants of approximately 6.25 hectares. Utilizing single-species and single-season occupancy models, we estimated an occupancy (*ψ*) of 0.21 and found that the presence of *P. olallae* groups was positively related to heterogenous plant composition forests which indicates they may be more likely to occur in areas of high floristic diversity. We also found that groups of this already range restricted species do not occur in all the forest coverage within their distributional range. This complements previous considerations regarding the habitat in which *P. olallae* occurs based on its natural history knowledge and highlights the limited suitable habitat for this species. From our sampling effort, we calculated a power of 81% to detect a population change of 30%, showing the potential of occupancy modeling for population monitoring of *P. olallae.* Thus, we provide an information baseline that will be useful in further population monitoring actions for the conservation of these endemic Bolivian titi monkeys.

## Introduction

Habitat loss is the most important threat to primate populations. Currently, more than 70% of primate species are considered threatened, with deforestation and forest fragmentation as the main risks due to increasingly intensive human activities (IUCN 2024). Other threats, such as hunting, climatic change, wildlife trafficking, and diseases augmented their relevance in the last years (Estrada et al. 2017; Fernandez et al. 2021; Barnett et al. 2024). Information about population trends and associated factors is crucial for making management decisions, and monitoring can provide this knowledge to prioritize conservation actions and assess their effectiveness (Keane et al. 2012; Fernandez et al. 2021). Traditional population monitoring methods focused on abundance metrics suffer from distinct issues related to primates’ behavior (e.g., motility and cryptical behavior conduct) that can compromise model assumptions, as well as the high costs and long time required when applied to large scales (Keane et al. 2012; Farris et al. 2019; Johnson et al. 2020). Moreover, although important biological and ecological information has been generated for many species in the last decade, there are groups such as titi monkeys for which the relationship between distinct habitat features and their distribution is not fully understood (Souza-Alvez et al. 2023; Barnett et al. 2024).

The Olalla’s titi monkey (*Plecturocebus olallae*) is Critically Endangered, endemic to Bolivia, and listed among the most threatened primate species across the world (Martinez and Wallace 2019, 2021a). This species is restricted to an area of 383.4 km^2^ in the Llanos de Moxos wetland, where the forest is naturally fragmented (covering only around 50% of the distributional range) but also significantly threatened, demanding monitoring efforts for this primate (Martinez and Wallace 2021a, 2021b; Wallace et al. 2013). Cattle ranching is the main economic activity in the region inhabited by *P. olallae*. This activity manages natural grasslands as pastures, with fire used annually to promote their regrowth, representing the most important and recurrent risk of habitat degradation and loss as fires can reach forest areas where these primates occur (Martinez and Wallace 2021b). In addition, a proposed intensification of human activities related to intensive agriculture (GADB 2019) would greatly increase the region’s biodiversity pressures. Previous research provided valuable knowledge on the biology, ecology, and population status for the conservation of *P. olallae* (Martinez and Wallace 2010, 2016a, b, 2019, 2021a, b; Wallace et al. 2013; Lopez-Strauss and Wallace 2015; Martinez et al. 2022), and currently, two municipal protected areas cover most of its distribution range and consider this species as a conservation priority (HCMSRY 2007; HCMSR 2019). Population monitoring is the next stage to apply suitable conservation measures for *P. olallae*, but a baseline of information on the distribution and demography, as well as their main drivers, is necessary for the development of and adequate tool able to detect population changes over time.

Originally used in other groups of vertebrates, occupancy modeling has been recently incorporated into primate research to obtain estimates of proportion of sites occupied by a species and is considered an adequate tool for monitoring their populations (Keane et al. 2012; Neilson et al. 2013; Sales et al. 2015a, b; Campbell et al. 2019; Johnson et al. 2020; Sushma et al. 2022). The inability to always detect a species when it is actually present at a site—referred to as imperfect detection—is a main source of bias from field data collection and occupancy modeling accounts for it enabling the obtention of accurate results which in turn can help increase the effectiveness of wildlife conservation actions (MacKenzie et al. 2006; Keane et al. 2012; Wallace et al. 2020). In addition, the ability to use data from rapid and short visits as well as from direct and indirect observations on the study species makes occupancy modeling a versatile tool for biodiversity monitoring and conservation, both in terms of time and budget (Johnson et al. 2020).

As a Callicebinae, *P. olallae* displays territorial behavior, which includes the emission of characteristic territorial calls that permit reliable detection of the species at a distance without direct observation (Martinez and Wallace 2016b). This territorial behavior allows the use of playback to promote the emission of territorial calls and thereby locate titi monkey groups, a technique that proved to be useful for the simultaneous detection of several groups (Martinez and Wallace 2007, 2021b; Lopez-Strauss and Wallace 2015). In addition, the relatively small home ranges (Martinez et al. 2022) provide an opportunity to use small spatial analysis units to obtain occupancy estimates for the species. The small distributional range of *P. olallae* (Martinez and Wallace 2021b) also suggests that most of its extension could be sampled for occupancy. These biological and ecological features of the Olalla’s titi monkeys suggest that suitable data on groups’ presence could be obtained to be analyzed using occupancy modeling and get reliable information to monitor their population.

Within its naturally fragmented forests habitat, the groups of *P. olallae* were not found in all the forest zones but occurred in areas with short trees (average canopy of 15 m) with a remarkable presence of vines and motacu palm (*Attalea* sp.), as well as terrestrial bromeliads (*Bromelia serra*) (Martinez and Wallace 2007; 2010; Wallace et al. 2013). This species consumes fruits and leaves from a variety of plant species that must be found across both seasons (Martinez et al. 2022) which, combined with its territorial behavior, suggests that groups might prefer areas of high plant diversity which offer enough food. Therefore, there is a need to know the suitable forest habitat for *P. olallae* which will be valuable for the design of effective monitoring and conservation actions for this threatened species.

In this paper, we present the results of an assessment of the occupancy modeling as a potential tool for the monitoring of *P. olallae* population. We considered distinct covariates that might help to identify the habitat features related to the presence of *P. olallae*. Based on the natural history knowledge on this species, we hypothesized that their presence would be conditioned by the forest type, expecting higher occupancy in forests of complex vegetation physiognomy (that would reflect a high variety of plant species), with high abundance of vines, and of short trees, whereas low occupancy values might correspond to areas with tall trees, low vine abundance and with a simpler physiognomy caused by a high abundance of fewer plant species. In addition, we assessed the feasibility to identify the suitable areas for *P. olallae* from the current remote information available on vegetation types. In this way, we aimed to provide baseline information to conduct permanent population monitoring for this threatened species.

## Methods

### Study area

**Fig. 1:**
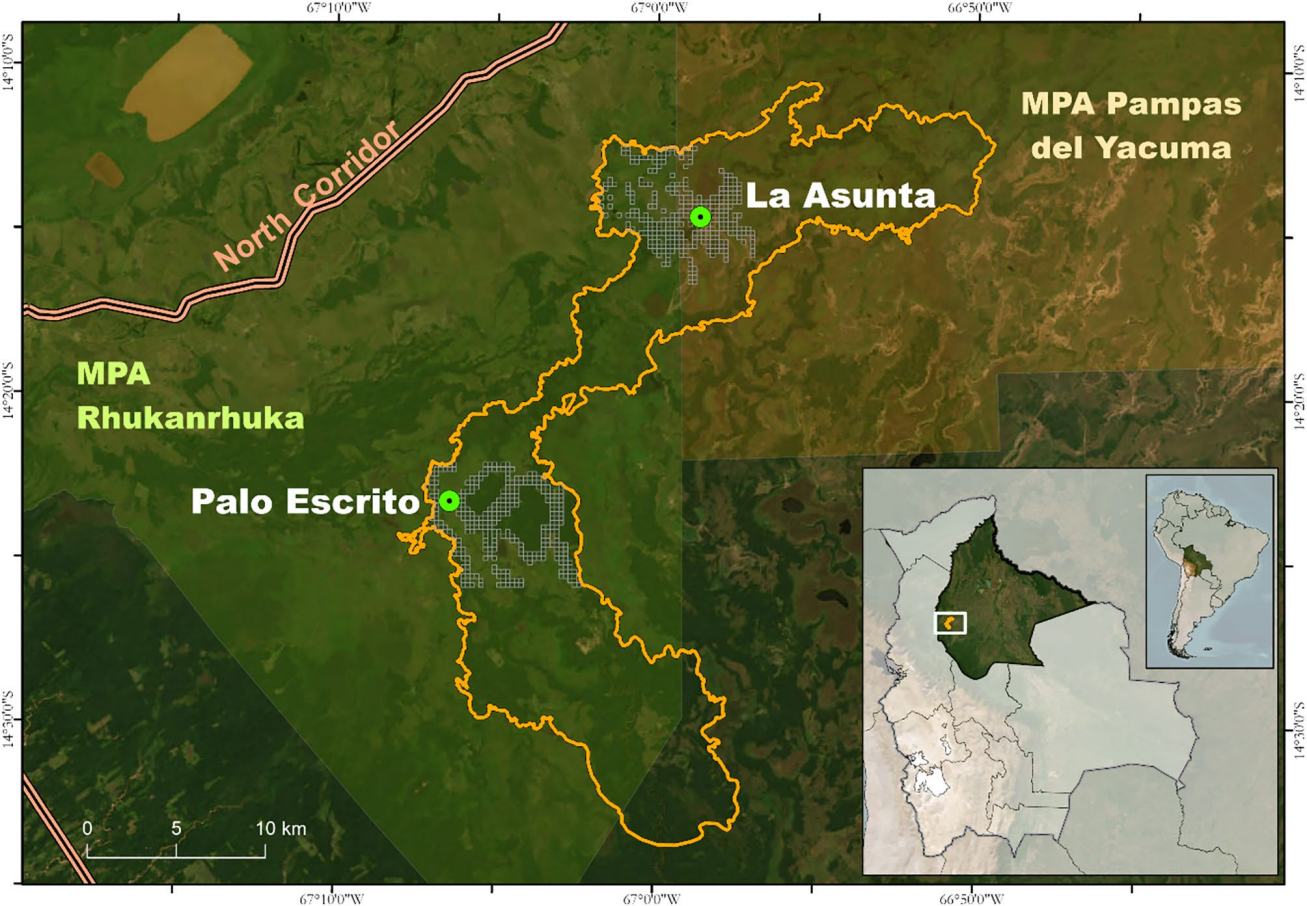
Location of the study sites at La Asunta and Palo Escrito, showing the grid system used in the design of the occupancy study for *Plecturocebus olallae*

We searched for groups of *P. olallae* during October 2019 in two areas: La Asunta (66° 58′ 40.32″ W; 14° 14′ 32.24″ S) and Palo Escrito (67° 6′ 25.27″ W; 4° 23′ 13.90″ S) cattle ranches and their surroundings. These two areas are in the largest forested areas across the range of *P. olallae*, and we selected them from previous knowledge on the distribution of this species (Fig. 1; Martinez and Wallace 2007, 2010, 2021b; Wallace et al. 2013). The forest coverage in the overall range of *P. olallae* is very low at < 50% (Wallace et al. 2013), and a remote assessment suggests even higher forest fragmentation levels with only around 23% of forest coverage and just three forest patches larger than 100 ha (Siles et al. 2019).

The region is part of the Llanos de Moxos wetland, characterized by a matrix of seasonally flooded lowland grasslands and forest patches (Hanagarth 1993). Cattle ranching is the main economic activity which manages grasslands as pastures and uses fire on an annual basis as a traditional method to promote grass regrowth (Martinez and Wallace 2007, 2010, 2016a, 2019, 2021a, b; Wallace et al. 2013). The extraordinary opportunities for wildlife observation have turned ecotourism into an important economic activity in the region and contributed toward the consolidation of two Municipal Protected Areas (MPA) in the municipalities of Santa Rosa del Yacuma and Reyes: Pampas del Yacuma and Rhukanrhuka, respectively (HCMSRY 2007; HCMR 2019). These two protected areas look to preserve the natural richness of the region and consider *P. olallae* as a conservation priority. La Asunta site is in the Pampas del Yacuma MPA, while Palo Escrito is in the Rhukanrhuka MPA.

### Playback sampling for P. olallae

We assessed the presence of *P. olallae* based on a grid system with 250 × 250 m cells overlapped with the entire distributional range of the species. The cell area (6.25 ha) is equivalent to the home range of *P. olallae* (7.20 ha; Martinez and Wallace 2016a; Martinez et al. 2022), and thus it represents an area that could be inhabited by a group of titi monkeys. To avoid grassland areas unsuitable for primates, we first selected grid cells with at least 75% of forest coverage according to satellite imagery data (IKONOS—Space Imaging 4-m MS and Global Mapper—Blue Marble Geographics). We selected 303 cells for La Asunta and 279 for Palo Escrito attempting to cover as much of the forest coverage as possible at each site.

**Fig. 2:**
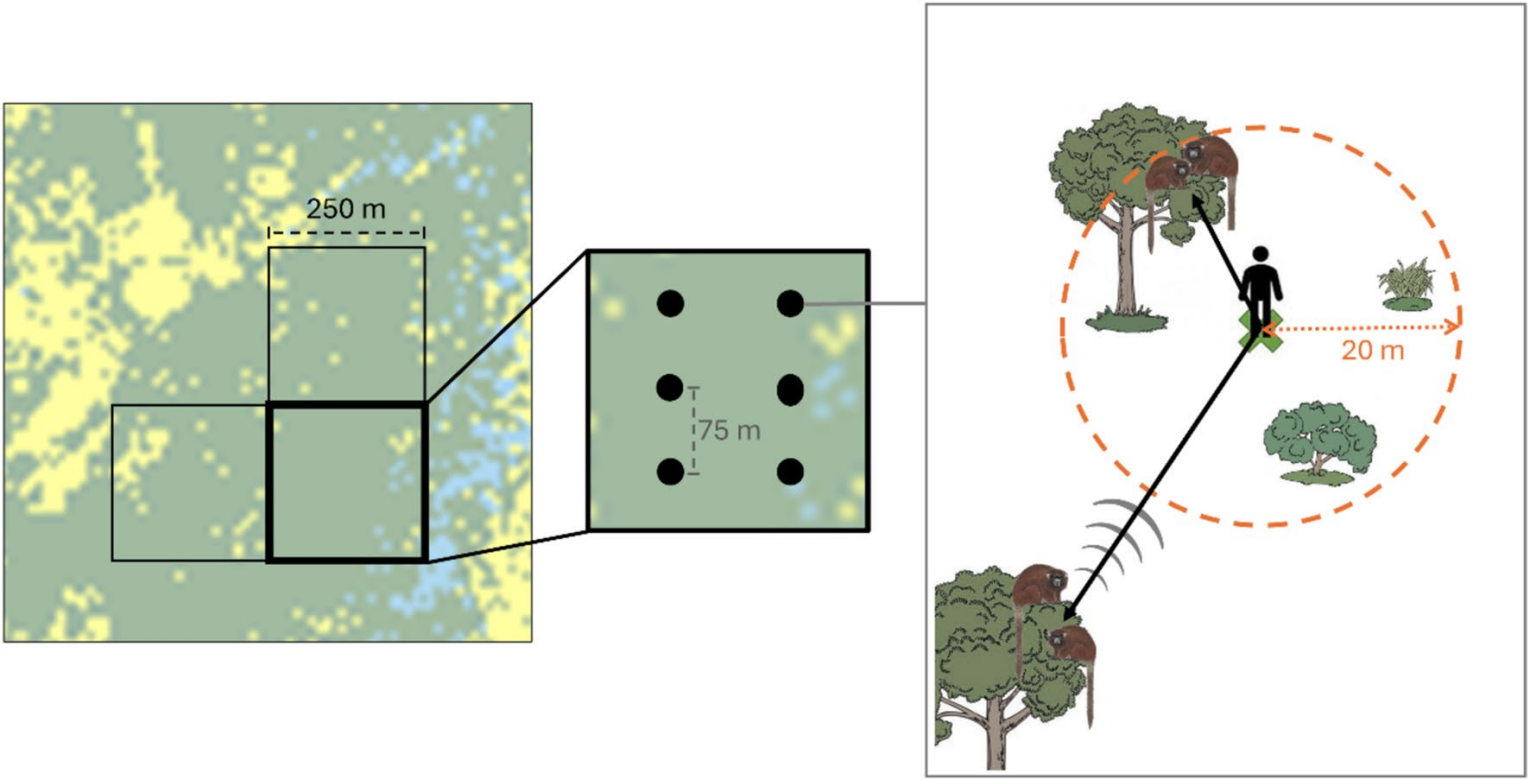
Schematic representation of the spatial sampling design, showing the location of point counts in the assessed grid cells and how data were collected on the location of heard and observed groups of monkeys, as well as habitat features

We conducted a rapid assessment of the presence of groups of *P. olallae*, performing one sampling of each selected grid cells by means of point counts where we used the playback technique of the territorial calls emitted by this species. Like Costa-Araújo et al. (2021), we conducted a previous pilot study to develop a sampling protocol to maximize the detection of groups of this territorial species (if present in a grid cell), considering that territorial calls and displacements occur within their territories as titi monkeys tend to avoid entering the areas of neighboring groups (Martinez and Wallace 2016b). Thus, we set six-point counts in each grid cell, separated by 75 m (Fig. 2), considering 250 m as the maximum effective distance at which titi monkeys could hear and respond to the playback (Martinez and Wallace 2007, 2021b; Lopez-Strauss and Wallace 2015; Sales et al. 2015a). At each point count, we performed a one-minute playback of territorial calls and waited for five minutes for any vocal or visual response of titi monkeys before moving to the next point count. We aimed to register information on the titi monkeys and their habitat (detailed below) in the five minutes window at each point count. From previous experience with the playback technique, we conducted the sampling from 06:30 to 12:30 h to maximize detection probabilities (Martinez and Wallace 2007, 2021b; Lopez-Strauss and Wallace 2015).

Titi monkeys responded to playback with the emission of territorial calls or by moving toward the observers in the point count place when they were close, allowing direct observations. We recorded the group location based on a compass bearing and visually estimated distance from the point count to the titi monkey calls or the first individual directly observed. As our sampling took place in the morning, when spontaneous territorial calls could be heard (Martinez and Wallace 2016b), we also collected the above bearing and estimated distance data for any titi monkey group emitting territorial calls. From direct observations, we registered the number of individuals of each group. At each point count, we registered our arriving time and georeferenced its location (Garmin GPSMAP 64 s).

We worked simultaneously in two teams of two people each, with each team assessing grid cells in zones assigned daily and separated by at least one kilometer to avoid any overlap from playback emissions. We found that some grid cells consisted of shrub dominated forest (canopy height between 2 and 3 m) with very few trees, which is an unsuitable forest habitat for titi monkeys (Martinez and Wallace 2007, 2021b), and we conducted two point counts in these cells to generate history detections and include them in the analysis. Following these logistics, we were able to assess around 12 cells/day per work team and then assess all the selected grid cells in 23 effective working days.

### Detection covariates

Previous knowledge on the vocal behavior of *P. olallae* indicates that territorial calls are emitted for several minutes and sometimes individuals can move toward the observers in response to playback (Martinez and Wallace 2007, 2016b; Martinez et al. 2022). As these reactions could promote positive detections in subsequent point counts from the same group of titi monkeys after the first detection, we calculated the Trap Affinity to be analyzed as a detection covariate to account for an overestimation of the number of groups (Hines J.E. com. pers.). This covariate assumes constant positive detections in all point counts of a grid cell after the first detection of titi monkeys and can be contrasted with the actual detection data to assess whether the first detection influenced further detections in subsequent point counts in a grid cell. Thus, we constructed a new matrix, considering the point counts where we made the first titi monkey detections for each cell, and filling in the information of all the subsequent counting points as having positive titi monkey detections. This simulated matrix was contrasted with our actual data on titi monkey group detections.

In addition, our experience with the playback technique showed us that it was effective to detect titi monkey groups at any time of the day (Martinez and Wallace 2007, 2016b, b; Lopez-Strauss and Wallace 2015). Then, although we conducted the sampling from early morning to noon, aiming to maximize detection probabilities, we considered the time of each point count as an additional detection covariate to assess any potential time-related bias.

### Local scale covariates

In a radius of 20 m from each point count, we recorded vegetation data to assess habitat variations for *P. olallae* at a local scale. We registered the visually estimated average canopy height, presence/absence of soil bromeliads, and the relative abundance of vines following a qualitative visual scale considering how much of trees were covered by them (high, medium, low, and absence, Mostacedo and Fredericksen 2000); all of them considered as potential indicators of the presence of titi monkeys (Martinez and Wallace 2007, 2010; Wallace et al. 2013). We also registered the presence/absence of grass, the presence/absence of water courses, and the presence/absence of forest burning signals in the vegetation (burnt trunks or stems, black color of tree bark, and presence of a layer of ash on the ground or plants).

Apart from the shrub forests with very few trees described above, we found areas with a noticeable large number of palm trees. We considered these two habitats as a proxy for the dominance of shrubs and palms and thus we named these areas as “palm forest” or “shrub forests”. By excluding these zones, we considered the remaining forest areas to have a more heterogenous plant composition and named them as “only forest”.

The above local habitat information was included as site covariates for each grid cell (Table 1, supplementary figure). For the categorical measurements, we determined the most representative feature in a grid cell based on the most common feature observed among the points counts performed in it, while we calculated average values for the continuous covariates. We disaggregated the distinct levels of each of the above local habitat features into separated presence/absence covariates (Table 1), which allowed us to obtain a better database structure for further analyses on the presence/absence of titi monkeys (MacKenzie et al. 2006).

**Table 1:** Covariates included in the occupancy modeling assessment for *Plecturocebus olallae*

Type	Group	Covariate name	Measurement	Collected in the field
Detection	Time		Time for each point count	x
	Trap affinity		Presence / absence	
Occupancy	Habitat	Forest	Presence / absence	x
		Grassland	Presence / absence	x
		Grassland—trees	Presence / absence	x
		Motacu palm forest	Presence / absence	x
		Palm forest	Presence / absence	x
		Shrub forest	Presence / absence	x
		River	Presence / absence	x
		Stream	Presence / absence	x
		Burned area	Presence / absence	x
		Only forest	Presence / absence	
	Bromeliads	Terrestrial bromeliads	Presence / absence	x
	Vine abundance	High	Presence / absence	x
		Medium	Presence / absence	x
		Low	Presence / absence	x
		No vines	Presence / absence	x
	Vegetation height	Average canopy height	Meters	x
	Altitude	Grid cell centroid altitude	Meters	
	Coverage	Forest	Coverage proportion	
		No forest (grassland)	Coverage proportion	
		Water	Coverage proportion	
	Vegetation type from available remote information	Mature forest	Coverage proportion	
		Successional forest	Coverage proportion	
		Herb coverage	Coverage proportion	
		Grassland	Coverage proportion	
		Flooded forest	Coverage proportion	
		Water	Coverage proportion	
		Human disturbed area	Coverage proportion	
	Distances to	Main roads	Meters	
		Secondary roads	Meters	
		Human settlements	Meters	
		Water bodies	Meters	
		Rivers	Meters	
		Nearest cell with titi monkeys	Distance in number of cells	
		Nearest cell with Only Forest	Distance in number of cells	

Covariates reliant on data collected in the field are marked to distinguish them from covariates based on remote data

### Landscape scale covariates

Attempting to find some indicator of suitable habitat for *P. olallae* based on the remote sensing information available, we used a preliminary unsupervised vegetation classification developed from high-resolution (Sentinel-2) satellite imagery which was available after our field campaign (Huarina 2022). This classification provided seven vegetation types which were contrasted with a national vegetation classification (Navarro 2011) and thus they were named as Mature forest: areas with a well consolidated arboreal community; Successional forest: secondary vegetation areas with short tree species; Herb coverage: non-arboreal plant species, with presence of herbs and shrubs; Flooded forest: seasonally flooded areas; Grassland: savannah habitats; as well as Water and Human disturbed areas. Although not yet validated in the field, this is the only remote habitat information for the range of *P. olallae* and we wanted to perform a preliminary assessment of their accuracy to identify areas inhabited by *P. olallae* by contrasting the habitat categories with our collected data on the presence of this species*.* In addition, as a proxy for the forest fragmentation, we utilized the forest coverage data previously used for the grid cell selection and included altitudinal and georeferenced information of roads and human settlements available from the Bolivian Geographic Database (GeoBolivia, https://geo.gob.bo).

From the above information, we generated landscape scale covariates for each grid cell (Table 1, supplementary figure) such as coverage proportion of forest, grassland or no forest, and water, as well as the coverage proportion of seven distinct vegetation types from the unsupervised classification. We calculated distances from grid cell centroids to distinct features, such as water bodies/courses, and to roads and human settlements as a proxy for human disturbance. We also considered the proximity of a grid cell to the nearest grid cell with presence of titi monkeys and to the nearest cell with the Only forest habitat (Distance to Nearest Cell with Presence of Titi Monkeys and Distance to Nearest Cell with Only Forest, respectively).

### Location of titi monkey groups and remotely identified vegetation types

Although the remote information on the vegetation types in the range of *P. olallae* was not yet validated in the field, we attempted to assess its correspondence with the location of the detected titi monkey groups. Nevertheless, as titi monkey groups detected only by their vocalizations were not observed, we did not know their exact location as occurred with directly observed groups. Hence, we estimated the location of the only heard groups with a triangulation based on the data collected on the distance and compass-bearing data for every group heard from each point count. We used the ‘Bearing Distance to Line’ tool (ArcMap v.10.8) to plot the distance and bearing data as lines, using line intersects to determine the location of the heard groups of titi monkeys. Thus, we generated a map of the location of all the groups detected in the field.

### Data analysis

We carried out the analysis in two stages, with the first stage assessing distinct occupancy models, including the entire set of covariates (Table 1), aiming to find the most relevant to the titi monkey occupancy. The second stage consisted of a sample size adjustment to obtain more representative results by discarding areas identified as not suitable for titi monkeys.

We used the PRESENCE software (Hines 2006; MacKenzie et al. 2006) to fit single-season occupancy models (MacKenzie et al. 2006, 2018), to estimate the probability of *P. olallae* occupancy, *ψ*, the detection probability parameter, *p*, and the influence of the factors that affect these parameters. We also calculated the Akaike Information Criterion (AIC) and Quasi Akaike Information Criterion (QAIC). We modeled detection at *i* = 1 to *S* sites during *j* = 1 to *K* visits as y_*ij*_ ~ Bernoulli (*Z*_*i*_ × *p*_*ij*_), where *Z*_*i*_ is the latent site-specific occupancy indicating whether the monkey is present in the cell (*Zi* = 1) or not (*Z*_*i*_ = 0), *p*_*ij*_ is the site/visit-specific detection probability, Pr(*Y*_*ij*_ = 1 | *Z*_*i*_ = 1), *S* is the number of sites (582 and 380 grid cells for stage 1 and 2, respectively) and *K* is the number of visits (point counts at each grid cell). We modeled the occupancy as *Z*_*i*_ ~ Bernoulli(*ψ*_*i*_), where *ψ*_*i*_ is the site-specific presence probability Pr(*Z*_*i*_ = 1).

To examine the influence of covariates on detection and occupancy, we used linear-logit models for each probability: logit(*p*_*ij*_) = *x*_*ij*_*α* and logit(*ψ*) = *x*_*j*_*β*, where *x*_*ij*_ is a vector of the variable that varies by site and visit, and α and β are the associated regression coefficients. To reduce the number of potential models, given the number of covariates (Table 1), we employed a variant of the two-step approximation for model fit (Karanth et al. 2011; Fuller et al. 2016). First, we conducted individual separated models for each detection and occupancy covariates to select those with a significant effect and the lower Akaike Information Criterion (AIC). Then, we tested our hypotheses by incorporating the detection covariates individually to select the best detection model. Thus, we fixed the best detection model and tested our hypothesis about forest habitat features affecting titi monkey presence by incorporating the occupancy covariates one by one. We ran a goodness of fit test for the models (10,000 bootstrap iterations) and corrected the AIC, for model selection, using the overdispersion parameter ‘ĉ’ to calculate the QAIC (MacKenzie and Bailey 2004; Kéry and Royle 2016). Based on the parameters of the best models selected, we assessed the power to detect a difference of 30% in the occupancy estimates considering the same sampling effort employed in our data collection campaign (Guillera-Arroita and Lahoz-Monfort 2012; Hines 2014).

Previously to test distinct covariates combinations for occupancy models, we assessed the correlation between covariates and found negative significant correlations (≥ 85%) only between forest and grassland proportion coverages (Spearman *r* = − 0.989, *p* < 0.001), which was expected due to the mutual opposite nature, as well as between distances to roads and human settlements (*r* = 0.880, *p* < 0.001). We did not include these correlated covariates in the same occupancy models tested to avoid over-parametrization.

Regarding the vegetation types and the location of titi monkey groups, we overlapped the remote information on the seven vegetation types described above with the locations of the groups directly observed as well as the estimated locations by means of triangulation for the only heard groups. Then, we obtained the number of titi monkey groups found for each of the vegetation types. In this way, we performed a preliminary assessment of the correspondence between the areas inhabited by the titi monkeys and the remote habitat information at landscape scale available so far.

## Results

We registered the presence of groups of *P. olallae* in 72 of the 582 grid cells assessed (35 in La Asunta and 37 in Palo Escrito), giving us a naïve occupancy estimate (proportion of grid cells with presence of titi monkeys) of 0.12. The occupancy estimation provided by the null model was *ψ* = 0.14 (SE = 0.02, 95% confidence interval: 0.12–0.18). We found that most of the grid cells marked with presence of *P. olallae* contained just one group, except five cases (7%) that had two groups.

We found three best performing models from the first analysis stage (Table 2), all including “Only Forest” as the strongest covariate which was positively related to the presence of *P. olallae* (Table 3). Another relevant covariate was the Distance to Nearest Cell with Only Forest, which was present in the two best models and showed a negative relationship with the presence of *P. olallae* (Table 3), while we found a similar negative relationship between the presence of *P. olallae* and the Distance to Nearest Cell with Presence of Titi Monkeys in the third best model (Table 3). We found a positive relationship between the presence of *P. olallae* and the Distance to Human Settlements (first and third model), whereas we found a negative relationship for the Distance to Main Roads (second model) (Table 3).

**Table 2:** Results of model selection showing the most relevant site (psi) and detection covariates (*p*) on the occupancy of *Plecturocebus olallae* from all the 582 sampled sites

Model	*K*	QAIC	ΔQAIC	AIC weight	Cumulative weight	QLogLik
psi(only forest + distance to nearest cell with only forest + distance to human settlements), p(trap affinity)	6	489.48	0	0.58	0.58	864.23
psi(only forest + distance to nearest cell with only forest + distance to main roads), p(trap affinity)	6	491.35	1.87	0.23	0.81	867.62
psi(only forest + distance to nearest cell with titi monkeys + distance to human settlements), p(trap affinity)	6	491.67	2.19	0.19	1.00	868.20

*K*: number of parameters

**Table 3:** Best *Plecturocebus olallae* occupancy models from all the 582 sampled sites, showing estimated probability of occupancy (psi [standard error and 95% confidence interval]), and detection probability (*p*, [standard error])

			Detection probability covariates		Occupancy covariates	
Model	Psi [SE, (95% CI)]	*p* [SE]	Covariate	β [SE]	Covariate	*β* [SE]
psi(only forest + distance to nearest cell with only forest + distance to human settlements), p(trap affinity)	0.15 [0.03, (0.10–0.21)]	0.39 [0.04]	Trap affinity	0.59 [0.23]	Only forest	1.96 [0.35]
					Distance to nearest cell with Only Forest	-1.25 [0.44]
					Distance to human settlements	0.64 [0.17]
psi(only forest + distance to nearest cell with only forest + distance to main roads), p(trap affinity)	0.14 [0.03, (0.10–0.21)]	0.39 [0.04]	Trap affinity	0.57 [0.23]	Only forest	1.86 [0.35]
					Distance to nearest cell with Only Forest	-1.22 [0.44]
					Distance to main roads	-0.56 [0.17]
psi(only forest + distance to nearest cell with titi monkeys + distance to human settlements), *p*(trap affinity)	0.15 [0.03, (0.10—0.21)]	0.39 [0.04]	Trap affinity	0.59 [0.23]	Only forest	2.14 [0.34]
					Distance to nearest cell with titi monkeys	-0.72 [0.26]
					Distance to human settlements	0.44 [0.18]

Estimated *β* values and standard errors are provided for each detection and occupancy covariate included

The second analysis stage was conducted with a subset of data (380 cells), not including the areas of shrub forest as this does not represent suitable habitat for titi monkeys. We found two best models in which the relationships between the presence of *P. olallae* and the covariates were similar to the first stage results, except by the Distance to Main Roads which was not present in the results of this analysis stage (Tables 4, 5).

**Table 4:** Results of model selection showing the most relevant site (psi) and detection covariates (*p*) on the occupancy of *Plecturocebus olallae* from the adjusted sample size of 380 sites

Model	*K*	QAIC	ΔQAIC	AIC weight	Cumulative weight	QLogLik
psi(only forest + distance to nearest cell with only forest + distance to human settlements), p(trap affinity)	6	643.48	0	0.69	0.69	833.56
psi(only forest + distance to nearest cell with titi monkeys + distance to human settlements), p(trap affinity)	6	645.17	1.69	0.30	0.99	835.78
psi(only forest + distance to nearest cell with only forest + distance to main roads), p(trap affinity)	6	651.63	8.15	0.01	1.00	844.31

*K*: number of parameters

**Table 5:** Best *Plecturocebus olallae* occupancy models from the adjusted sample size of 380 sites, showing estimated probability of occupancy (psi [standard error and 95% confidence interval]), and detection probability (*p*, [standard error])

			Detection probability covariates		Occupancy covariates	
Model	Psi [SE, (95% CI)]	*p* [SE]	Covariate	*β* [SE]	Covariate	*β* [SE]
psi(only forest + distance to nearest cell with only forest + distance to human settlements), p(Trap affinity)	0.21 [0.04, (0.14–0.29)]	0.40 [0.04]	Trap affinity	0.52 [0.22]	Only forest	1.30 [0.36]
					Distance to nearest cell with Only Forest	-0.94 [0.40]
					Distance to human settlements	0.81 [0.16]
psi(only forest + distance to nearest cell with titi monkeys + distance to human settlements), p(Trap affinity)	0.21 [0.04, (0.14–0.29)]	0.40 [0.04]	Trap affinity	0.52 [0.22]	Only forest	1.43 [0.35]
					Distance to nearest cell with titi monkeys	-0.64 [0.28]
					Distance to human settlements	0.67 [0.17]

Estimated *β* values and standard errors are provided for each detection and occupancy covariate included

All the best detection models we found in both analysis stages included trap affinity as a detection covariate. This reveals that when we detected titi monkeys in a point count, there was a higher chance to detect them in the subsequent ones within each grid cell. On the other hand, we found that the time of point counts was not a meaningful detection covariate. According to the parameters of the models from the second analysis stage from the adjusted sampled size (*N* = 380, *ψ* = 0.21, *p* = 0.40), we estimated a power of 81% to detect a change of 30% in the occupancy of *P. olallae* with a significance level of 0.2.

**Fig. 3:**
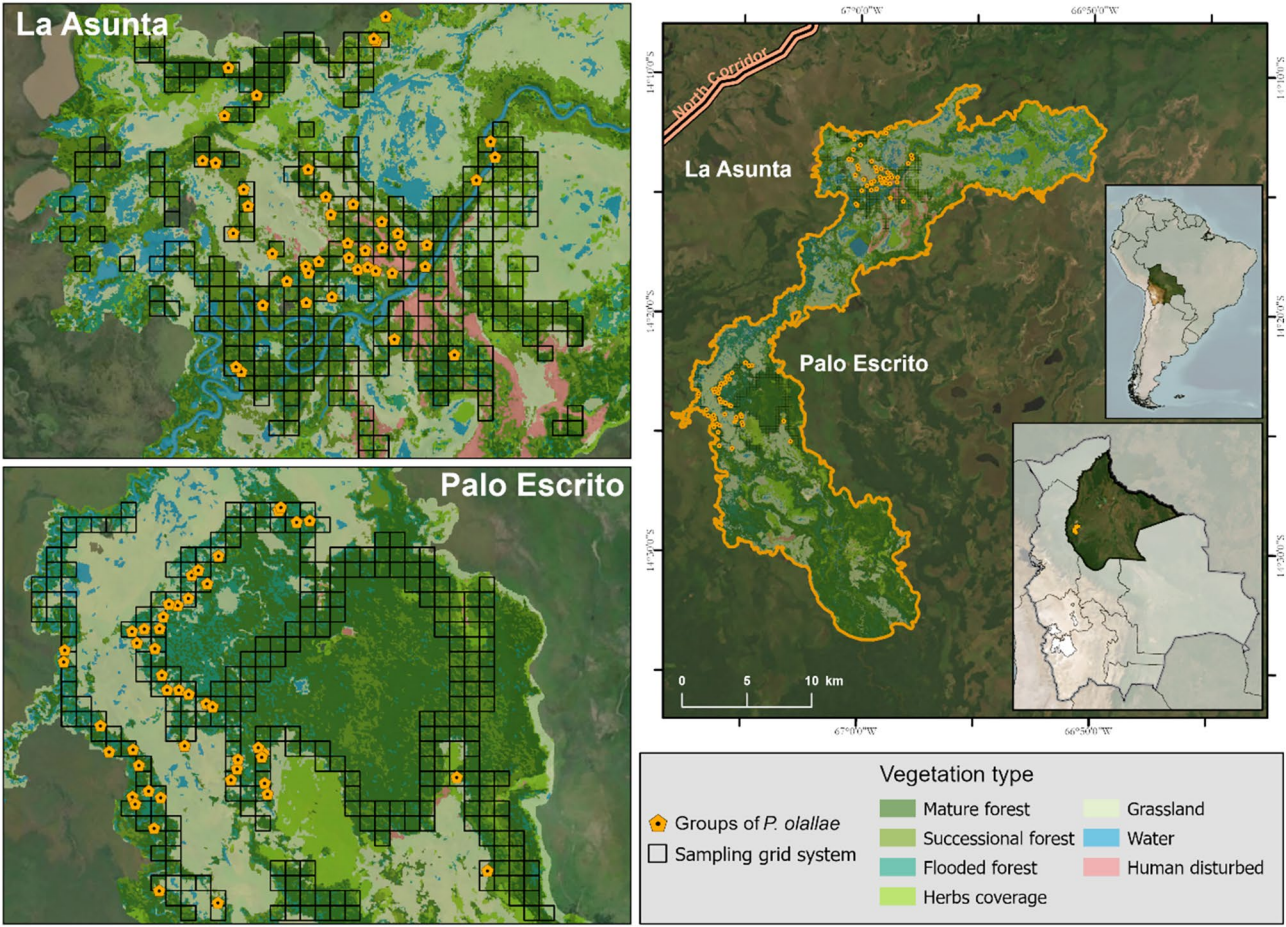
Location of *Plecturocebus olallae* groups based on triangulation of territorial calls and direct observation in relation with the remote information on vegetation types across the distribution range of the species

From direct observations of titi monkeys and the triangulation of territorial calls, we determined the location of 90 groups of *P. olallae*, 86 of them were in the grid system sampled (55 observed directly and 31 located by their vocalizations), and four groups were located outside the sampled grid cells. According to the remote data on vegetation types, we found that most of the titi monkey groups were in areas classified as mature forest (41%), as well as in successional forest (21%) and flooded forests (20%) (Table 6). Mature and successional forests were the most extensive vegetation types in the two study sites, but titi monkey groups were not found in all these areas (Fig. 3).

**Table 6:** Number of detected groups of *Plecturocebus olallae* according to vegetation types remotely identified

Vegetation type	Groups inside grid system	Groups outside grid system	Total
Mature forest	36	1	37
Successional forest	17	2	19
Flooded forest	18		18
Grassland	8		8
Herb coverage	4	1	5
Water	2		2
Human disturbed area	1		1
			90

## Discussion

We attempted to identify habitat features that might explain the presence of groups of *P. olallae* considering the canopy-dependence of Callicebinae (Bicca-Marques and Heymann 2013; Sales et al. 2015a) as well as the ecology and previous suggestions on habitat preference of this species (Martinez and Wallace 2007, 2010; Wallace et al. 2013; Martinez et al. 2022). As expected, the occupancy modeling results show that the presence of groups of *P. olallae* was mainly related to areas of heterogeneous plant composition (only forest covariate), suggesting these titi monkeys tend to inhabit areas with a relatively high floral diversity. We also found that the proximity to these heterogeneous plant areas or to areas with already confirmed presence *P. olallae* groups were also meaningfully related to the presence of this species, whereas it seemed less likely to be found in areas close to human settlements. On the other hand, we found that forest height and vine abundance were not meaningfully related to the presence of these primates as we expected from previous distribution information. Although the presence of *P. olallae* was not found to be meaningfully related to any remote covariate, preventing the extrapolation of occupancy results to its entire range, occupancy modeling seems to be an adequate tool to assess the demographic and range dynamics of *P. olallae*.

Our findings regarding the relevance of forest habitats with a heterogenous plant composition (only forest habitat covariate) on the presence of *P. olallae* provide support to previous insights about the habitat requirements of this species. These territorial primates exhibited a diet shift to alternative foods such as leaves during the fruit lean season while reducing movements, reflecting a clear dependence on the food productivity in their territories (Martinez et al. 2022). Therefore, these titi monkeys seem to prefer areas with a high flora richness which offer them enough food resources continuously, as opposed to zones where a marked dominance of any plant species might imply an irregular food offer across seasons as could happen with the areas where we found a high number of palms. In addition, considering the canopy-dependence of Callicebinae (Bicca-Marques and Heymann 2013; Sales et al. 2015a), shrub areas with very few trees would represent unsuitable habitat for the titi monkeys as they would not provide suitable protection sites for resting, sleeping, or hiding from predators. Thus, our second analysis stage in which we excluded the shrub forests as they do not fulfill the basic habitat requirements for *P. olallae* provides more representative results, also suggesting that further assessments might not need to consider the shrub forests but could redirect sampling effort to tree forest areas.

Previous distribution knowledge showed that groups of *P. olallae* did not occur in all the forest coverage across their range (Martinez and Wallace 2007, 2010; Wallace et al. 2013). Our occupancy modeling results show that groups of *P. olallae* were more likely to be found in areas close to the “only forest” habitat as well as to areas with confirmed presence of these titi monkeys. Moreover, our data on the location of titi monkey groups (from direct observations and call triangulation) showed that titi monkey groups do not occur throughout the forest extent. These results suggest a kind of clustered distribution pattern of the titi monkey groups, which might reflect not only the fragmented forest landscape where *P. olallae* occurs (Martinez and Wallace 2007, 2010; Wallace et al. 2013), but also the existence of a particular vegetation composition in specific parts of forest patches. Sales et al. (2015b) found that habitat continuity, represented by canopy connectivity, is a major determinant of the presence of black-fronted titi monkey *Callicebus nigrifrons* showing the importance of the extent of areas with suitable habitat in a region of fragmented forests. Then, the signals of a spatial clustering we found on the distribution of *P. olallae* groups show that suitable habitat may occur in specific areas which would represent an additional restriction factor to the previous distribution knowledge on this species (Martinez and Wallace 2021b). These potential local population isolations will need to be considered in future conservation actions.

We found that the distance to human settlements (villages and the Santa Rosa main town), was the only landscape scale covariate included in the best occupancy models, with a reduced presence of the titi monkeys nearer to human settlements that suggests negative effects on the titi monkey population. Nevertheless, it needs to be considered that human settlements are outside the study area and even outside the distributional range of *P. olallae*, then human settlements would not represent a direct risk of habitat loss for *P. olallae* although that is the main conservation risk for the species (Martinez and Wallace 2021b). In addition, the information on conservation threats does not mention considerable hunting pressure on *P. olallae* (Martinez and Wallace 2007, 2021a; Wallace et al. 2013). These considerations provide some support that distance to human settlements was not the most relevant covariate in the best occupancy models obtained. On the other hand, distance to the major road (an interstate route) was another landscape scale covariate present in the results, but only for the first stage of analysis, showing a higher presence of titi monkeys in areas closer to roads. In this case, we must consider the fact that forests occur in higher areas (Hanagarth 1993), where roads are also built to prevent damage by flooding. But the road was also outside the distribution range of *P. olallae* which might have reduced the relevance of the respective covariate in the final occupancy modeling results.

Regarding the detection of titi monkey groups, the similar naïve and estimated occupancy values also suggest that we detected most titi monkey groups present in the sampling areas. We also found that the detection of titi monkey groups was not affected meaningfully by the time of the day as the time detection covariate did not appear in the best occupancy models obtained. This shows the efficiency of our sampling between morning and noon using the playback technique, as has already been reported for other Callicebinae (Dacier et al. 2011: white-tailed titi monkey *Plecturocebus discolor*, Caselli et al. 2015; Sales et al. 2015a: *C. nigrifrons*, Martinez and Wallace 2007, 2021b: Beni titi monkey *P. modestus* and *P. olallae*). In addition, the meaningfulness of the trap affinity as a detection covariate in the best occupancy models confirms that the first detection of a titi monkey group promoted positive detections in the following point counts within a grid cell. This suggests that it may be feasible to cease playback after the first detection and leave a grid cell, such as in a *C. nigrifrons* occupancy assessment (Sales et al. 2015a), and use the time saved to assess other areas. Nevertheless, by completing the six established point counts per grid cell independently from positive detections of titi monkeys, we aimed to detect all the titi monkey groups in a grid cell, while also obtaining additional location data from multiple point counts for the triangulation of territorial calls as well as habitat data. These considerations can be important for further similar assessments on *P. olallae*.

Our assessment of the preliminary remote information on vegetation types in the range of *P. olallae* showed in the first instance that none of the seven categories was relevant for the occupancy modeling analysis. Moreover, although most of the locations of titi monkey groups were in a specific category (mature forest), they were not found throughout this vegetation type despite its great extension. Moreover, we also found some groups in areas remotely identified as grasslands, herb coverage, and water, showing further differences between remote and field-based information. Thus, local-scale habitat covariates appear to be more relevant on the presence of *P. olallae* than landscape scale habitat covariates as has also been reported for *C. nigrifrons* in fragmented forests (Sales et al. 2015a), suggesting that some habitat features important for *P. olallae* might be masked by the forest canopy. Therefore, more accurate work is required to recognize vegetation variations across the range of *P. olallae* through the inclusion of extensive in situ data on flora to validate and adjust the remote information tool aiming to a spatial extrapolation of occupancy modeling results.

The use of occupancy modeling is increasing across different primate species, but it is not frequently used for population monitoring yet (MacKenzie et al. 2006), as their general long lifespan and low reproduction rates difficult the estimation of minimum population sizes, which is a key reference to apply different conservation measures (Elzinga et al. 2001; Lindermayer et al. 2013). Despite the recent research advances for several Callicebinae species, long-term research is still required to acquire the necessary information on the population dynamics and promote increasingly more effective conservation and monitoring actions (Bicca-Marques and Heymann 2013; Sales et al. 2015b; Souza-Alves et al. 2023; Barnett et al. 2024). Thus, the design of monitoring tools with their respective effectiveness estimates represents valuable and urgent information while knowledge on species’ natural history is being increased (Lindermayer et al. 2013). Although more information on the population dynamics is still missing for *P. olalle*, our sampling effort might permit the detection of a 30% population change with a power of 81%, which seems an adequate power value for monitoring actions, allowing us to detect a significant, but not too abrupt, population change. Complementarily, the implementation of occupancy modeling demands a feasible detection probability, manageable logistics of sampling, and low risks of groups overcounting (Keane et al. 2012; Neilson et al. 2013). Our assessment of many sampling units, covering most of the distributional range of *P. olallae* in less than a month, shows the advantages of occupancy modeling to reduce bias from population changes. In addition, based on the loudness and characteristic structure of the territorial calls of *P. olallae* (Adret et al. 2018), there is a potential to automatize the data collection of groups presence by means of passive acoustic monitoring (Kalan et al. 2015) which could open new research line for the population monitoring of this threatened primate species.

We generated valuable information on *P. olallae* occupancy considering distinct habitat features, providing a baseline of information for further population monitoring of this endemic and Critically Endangered species. Despite some limitations in the use of the available remote vegetation data, we proved the feasibility and the potential of occupancy modeling as a monitoring tool for Olalla’s titi monkeys, while identifying aspects that could improve its usefulness. Our findings on habitat restrictions for *P. olallae*, which are not present in all forest habitats, emphasize the conservation importance of forest coverage to ensure population connectivity for these primates (Syxaiyakhamthor et al. 2019), and assist in prioritizing future monitoring tasks to ensure the efficiency of conservation actions for *P. olallae* and its habitat.

## Data Availability

The datasets generated during and/or analysed during the current study are available from the corresponding author on reasonable request.
